# Unveiling the Microbiota: A New Frontier in Breast Cancer Pathogenesis—A Single-Center Preliminary Study

**DOI:** 10.3390/diagnostics15172147

**Published:** 2025-08-25

**Authors:** Rukie Ana Maria Ahmet, Andrei Gabriel Nascu, Georgiana Cristina Camen, Cosmin Vasile Obleaga, Dragos George Popa, Cecil Sorin Mirea

**Affiliations:** 1 Doctoral School, University of Medicine and Pharmacy of Craiova, 200349 Craiova, Romania; 2 Department of Informatics, Faculty of Science, University of Craiova, 200585 Craiova, Romania; 3 Department of Radiology and Medical Imaging, Faculty of Medicine, University of Medicine and Pharmacy of Craiova, 200349 Craiova, Romania; 4 Department of Surgical Semiology, Faculty of Medicine, University of Medicine and Pharmacy of Craiova, 200349 Craiova, Romania; 5 Department of Plastic and Reconstructive Surgery, Faculty of Medicine, University of Medicine and Pharmacy of Craiova, 200349 Craiova, Romania

**Keywords:** microbiota, breast cancer, dysbiosis, artificial intelligence

## Abstract

**Background:** Breast cancer is the most common malignancy affecting women worldwide and continues to pose significant challenges despite progress in early detection and personalized therapies. While its pathogenesis has traditionally been associated with genetic, hormonal, and environmental factors, recent studies have highlighted the potential role of dysbiosis—an imbalance in gut and systemic microbiota—in breast cancer development and progression. This article aims to examine the mechanisms through which systemic dysbiosis may contribute to breast cancer risk and explore its therapeutic implications. **Methods:** This study seeks to analyze and compare the fecal microbiota profiles of breast cancer patients and healthy individuals from a single center in Craiova, Romania, in order to identify microbial signatures linked to breast cancer and BRCA mutation status. Special attention is given to the gut–liver axis and its influence on estrogen circulation, a key factor in hormone-sensitive breast cancers. **Results:** Evidence suggests that dysbiosis can influence breast cancer progression by promoting chronic inflammation, impairing immune regulation, and altering estrogen metabolism through the gut–liver axis. These effects may contribute to tumor development, immune evasion, and therapeutic resistance. Interventions aimed at restoring microbial balance show promise in preclinical studies for mitigating these effects. **Conclusions:** Systemic dysbiosis represents a potentially modifiable risk factor in breast cancer. Microbiota profiling may serve as a useful biomarker for risk stratification and therapeutic response. Future research into microbiome-based interventions could offer novel approaches for prevention and treatment in breast cancer care.

## 1. Introduction

Breast cancer represents a significant global public health challenge, currently recognized as the most commonly diagnosed malignancy among women worldwide. According to WHO, breast cancer is the most common cancer in women in 159 countries, out of 185 in the year of 2022, ranking as the fourth most common cause of mortality worldwide [1,2]. Despite advances in early detection, personalized medicine, and improved treatment regimens, it continues to pose significant morbidity and mortality rates. Our understanding of breast cancer pathogenesis has traditionally focused on genetic, hormonal, and environmental factors. However, an emerging body of evidence is unveiling a new potential player in the disease’s development and progression: the microbiota. The gut microbiota exerts a critical influence on human health through its roles in immune modulation, metabolic regulation, and the maintenance of inflammatory homeostasis. Disruptions to this microbial equilibrium, known as dysbiosis, have been increasingly associated with the pathogenesis of various malignancies, including breast cancer (BC). However, the interplay between the gut microbiome and breast cancer progression—particularly among individuals harboring BRCA mutations—remains insufficiently elucidated. This study seeks to characterize the fecal microbiota composition of breast cancer patients in comparison to healthy controls, with the objective of identifying microbial signatures associated with both breast cancer and BRCA mutation status.

## 2. Materials and Methods

### 2.1. Methodology and Data Acquisition

This retrospective case–control study was conducted at the University of Medicine and Pharmacy of Craiova between October 2020 and June 2025. A total of 99 women were included: 57 breast cancer patients and 42 healthy controls, including individuals with benign breast lesions and those with no detectable abnormalities. Participants were categorized as residing in either an urban or rural area based on the official administrative-territorial classification of Romania. According to this framework, urban areas were defined as municipalities and cities. Rural areas were defined as communes, which include the villages they are composed of. This clear delineation allowed for a consistent classification of participants and enabled a more robust analysis of the potential differences in gut microbiota composition related to geographic residence. In Romania, the administrative-territorial framework defines municipalities, cities, communes, and villages based on population size, economic importance, and infrastructure [3]. Inclusion criteria were women over 18 years old, including patients with undetected or benign breast lesions and newly diagnosed breast cancer patients who had not received antibiotics within one month prior to fecal sampling or who had not started chemotherapy or hormonal therapy. Exclusion criteria included acute or chronic gastrointestinal conditions within the last two months, mental disorders, and prior initiation of oncological therapy. The study protocol was approved by the Ethics Committee of the University of Medicine and Pharmacy of Craiova (Approval Code: 199/20 September 2023).

All participants provided written informed consent. The study did not interfere with ongoing medical care, and no experimental treatments were administered. Demographic, clinical, imaging, and pathological data were collected. This included pre-oncological evaluations, oncological and surgical treatments, and histopathological and immunohistochemical findings. Fecal samples were collected using sterile collection kits and stored accordingly. Samples were analyzed for pH, presence of proteolytic and protective bacteria (e.g., *Lactobacillus*, *Bifidobacterium*, *Enterococcus*), and fungi. Microbial DNA was extracted and subjected to 16S rRNA gene sequencing for comprehensive profiling. The V3–V4 regions of the 16S rRNA gene were amplified and sequenced. Alpha diversity (within-sample diversity) and beta diversity (between-sample diversity) metrics were calculated. Taxonomic composition was assessed at the phylum and genus levels, with comparisons between breast cancer patients and healthy controls.

### 2.2. Statistical and AI Data Modeling

The graphics and statistical analysis for data interpretation were performed using Python programming language scripts. We have chosen to utilize Python version 3.9.12 in an Anaconda Distribution for an easy way to manage packages in Python, and as the Integrated Development Environment, we utilized Jupyter Notebook v6.4.8.

All the graphics were performed by employing data handling, scientific computing, data visualization and artificial intelligence modules from python such as pandas, scipy, seaborn, sklearn and matplotlib.

For the collected database, a set of visual and statistical tests were made to ensure that a Gaussian distribution is followed for the only numerical feature, namely age. For an in-depth visual representation, two graphs were made:

- A histogram with one curve for a smooth kernel density estimation of the real data and a curve for the theoretical normal/Gaussian distribution based on the mean and standard deviation of the age in our dataset;
- A quartile to quartile plot to compare each instance (patient age) represented by a dot in the graphic with the normal distribution represented by a line.

To complement and strengthen the visual interpretation of the results, we added a Shapiro–Wilk test [4] with two hypotheses:

○ Null hypothesis ($H_{0}$): data are normally distributed;
○ Alternative hypothesis ($H_{1}$): data are not normally distributed.

**Figure 1:**
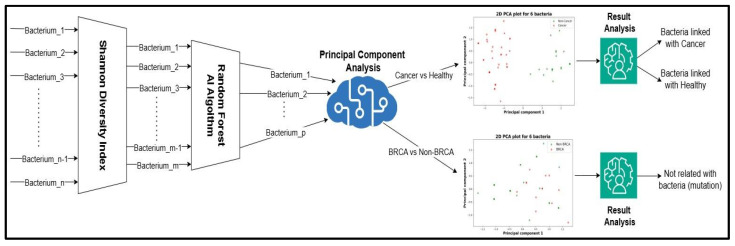
Proposed workflow for analyzing bacterial gut microbiota in relation to breast cancer. In the left part of image 2, dimensional reduction modules are presented first to eliminate bacteria with little to no change in patient analysis and second to eliminate bacteria that are not relevant for classifying cancer vs. non-cancer patients by Random Forest algorithm. In the right part of the image, a PCA algorithm is used to offer a visualization of the bacterial composition of the microbiota in a 2D space.

The workflow of the paper is described in Figure 1, which was made in draw.io. Firstly, on the n bacteria, a Shannon diversity index (SDI) filter was applied, resulting in m bacteria. The purpose of utilizing the SDI technique is to quantify the diversity of data for each of the initial bacteria. A higher value for the Shannon index implies an increased diversity for the analyzed bacteria [4]. To calculate the diversity for each bacterium, the following formula was employed:

$$H\;=\;-\sum_{i=1}^{n}p_{i}\;x\left({{log}_{2}(p}_{i})\right)$$

where H represents the Shannon diversity index for one bacterium,

n represents the number of different categories that one bacterium can have; in our study, each bacterium value can be low, medium, or high, so n will be 3.

$p_{i}$ represents the proportion of that category from the total recorded data.

The next action taken in the process of discerning which are the most relevant bacteria related to breast cancer diagnosis was to apply a Random Forest Machine Learning Algorithm [5] to reduce the dimensionality from m bacteria to p bacteria. This dimensionality reduction is performed by taking into consideration the impact of each bacteria on the decision making process of the algorithm; from this, those that are close to 0 will be eliminated.

On the resulting p bacteria, a Principal Component Analysis (PCA) algorithm was employed to better visualize the potential relationships for two main cases:

- Cancer and non-cancer patients;
- BRCA and non-BRCA patients.

PCA is a technique of dimensional reduction that can transform a p-dimensional space into a two- or three-dimensional space by creating principal components (PCs) based on the p bacteria. These components would be expressed like a linear combination of the p bacteria types, and based on the position of the cancer and non-cancer patients in the graphic, it will be concluded if the two sets can be linearly separable. Based on the PCA visualization, we draw a few conclusions. To increase the degree of confidence in the obtained results and interpretations from the visual representation given by the PCA, a statistical experiment is proposed. The experiment consists of two AI models of logistical regression:

○ Null model that does not know anything about gut microbiota;
○ Complete model that trains and sees the data about the bacteria composition for both cancer and non-cancer patients.

For this experiment, two statistical hypotheses arise:

○ Null hypothesis ($H_{0}$) which states that the complete model is not better that the null model. If true, this would mean that gut microbiota has no relationship with the presence of cancer.
○ Alternative hypothesis ($H_{1}$) which states that the complete model is significantly better than the null model. If true, this would mean that there is a relationship between cancer and gut microbiota.

## 3. Results

We conducted a retrospective study to comprehensively assess the microbial community in fecal samples. The research was carried out between October 2020 and June 2025 at the University of Medicine and Pharmacy of Craiova. A total of 99 participants were included, divided into two groups of 57 breast cancer patients and 42 healthy controls, encompassing individuals with benign conditions and those with no detected lesions.

**Figure 2:**
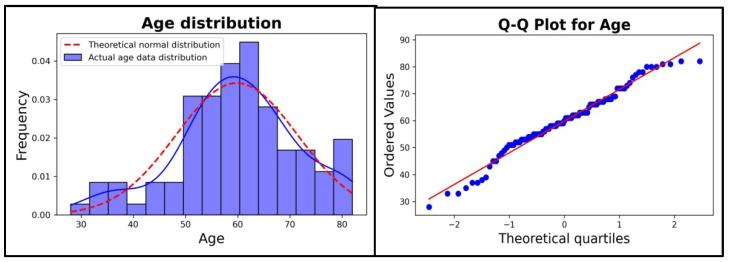
Age distribution analysis. Left: Histogram with both theoretical normal distribution and actual distribution of the data. Blue line suggests kernel density estimation of real data. Red line for the theoretical normal distribution based on the mean and standard deviation of the age dataset. Right: Quartile to quartile plot with normal distribution depicted as a line and actual patient values as dots Red line for the theoretical normal distribution based on the mean and standard deviation of the age dataset.

In Figure 2 Left, a histogram with the ages of the patients is plotted, and a blue line was added for a smooth kernel density estimation of real data and a red line for the theoretical normal distribution based on the mean and standard deviation of the age dataset. By comparing these two curves, a good enough level of similarity can be seen with a higher difference in the two curves on the left side. For further visual analysis, a quartile to quartile plot is shown in Figure 2 Right; depending on how well the blue dots align with the red line, the likelihood of a normal distribution can be visually quantified. As it can be observed from the image, there are a few blue dots on the ends of the line, especially on the left part, that do not fit very well with the Gaussian distribution.

A Shapiro–Wilk test was performed for a numerical verification of the Gaussian distribution for the age spectrum of the patients, where values of 0.976 for statistics and 0.064 for *p*-value were obtained. It can be concluded that we do not have sufficient proofs to deny the statement from the null hypothesis ($H_{0}$) because the *p*-value is above a standard threshold of α = 0.05, so the data follows approximately a normal distribution.

Data analysis revealed that among the 57 breast cancer patients, 13 were BRCA mutation carriers, while the remaining 44 did not exhibit a BRCA mutation. Of the BRCA mutation carriers, only one patient was 37 years old, with the remaining patients being over 50 years of age, predominantly from urban areas. Specifically, among the non-BRCA carriers, 38 were from urban areas, and 8 of the BRCA carriers also resided in urban settings (Table 1).

In Table 2, we presented a detailed profile of the BRCA carriers, assessing age, family history of breast cancer, diagnostic procedure, histogenetic type, histopathological diagnosis, and molecular classification.

The BRCA carrier groups in our study displayed distinct profiles. BRCA1 carriers were predominantly over the age of 40 and typically had a family history of breast cancer (BC). Diagnosis often occurred through clinical examination combined with biopsy, revealing malignant breast lesions, primarily invasive carcinomas of either ductal or lobular type. These tumors were commonly triple-negative.

In contrast, BRCA2 carriers were all over 40 years old, with no family history of BC. Their diagnoses were often made following clinical examination, a surgical procedure, and histopathological analysis. The breast lesions were invariably malignant, presenting as invasive carcinomas of either ductal or lobular type, and were most commonly luminal A-type tumors.

These subgroup differences, along with previously noted distinctions between BRCA carriers and non-carriers, align with findings reported in the literature. However, many of the key correlations in our study (e.g., age, family history, and histopathological diagnosis) were trend-like rather than statistically significant, likely due to the small sample size. This limitation stems from our center’s recent establishment and the exploratory nature of this study.

Among breast cancer patients, urban residency was associated with more pronounced microbial dysbiosis. Additionally, BRCA carriers tended to have a distinct microbial signature compared to non-carriers, suggesting a potential interaction between genetic predisposition and gut microbiota composition.

In Table 3, we presented a detailed profile of the non-BRCA breast cancer patients, assessing age, family history of breast cancer, diagnostic procedure, histogenetic type, histopathological diagnosis, and molecular classification.

Applying SDI on the dataset resulted in two clear separable bacterial groups as follows:

1. The bacteria with blue nuances of colors have a height diversity index;
2. The bacteria with yellowish nuances have a low to 0 diversity index which means an almost absence of variance in the data for those bacteria.

**Figure 3:**
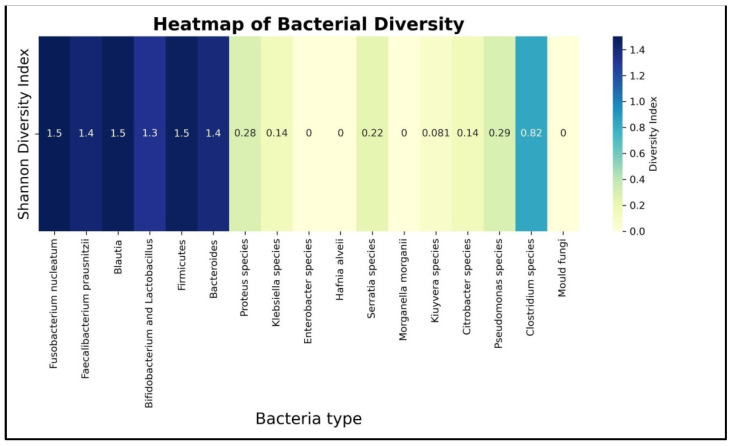
Heatmap of Shannon diversity index of all analyzed bacteria with colors assigned according to the SDI value.

Initially, 18 bacterial species were analyzed and, based on the Shannon index, it was observed that some of them were not relevant, as indicated by the heatmap in Figure 3. Consequently, a decision of exclusion was taken for those highlighted in yellow: *Proteus* species, *Klebsiella* species, *Enterobacter* species, *Hafnia alveii*, *Serratia* species, *Morganella morganii*, *Kluyvera* species, *Citrobacter* species, *Pseudomonas* species, *Clostridium* species, and *Mold fungi*. Subsequently, the focus of analyzing data moved from an initial 18 species to a total number of 7 species with a Shannon index greater than 0.4, which are highlighted in blue: *Fusobacterium nucleatum*, *Faecalibacterium prausnitzii*, *Blautia*, *Bifidobacterium* and *Lactobacillus*, as well as members of the *Firmicutes* and *Bacteroides phyla.*

Findings revealed that BC patients exhibited reduced microbial diversity compared to healthy controls. Specifically, there was a relative enrichment of *Firmicutes*, particularly *Clostridium clusters XIVa* and *IV*, in BC patients. Conversely, *Bacteroidetes phylum members*, including genera such as *Bifidobacterium*, *Odoribacter*, *Butyricimonas*, and *Coprococcus*, were depleted in the BC cohort. These alterations suggest a dysbiotic gut microbiota in BC patients, characterized by an overrepresentation of certain *Firmicutes* and a reduction in beneficial *Bacteroides*, which may influence breast cancer development and progression.

**Figure 4:**
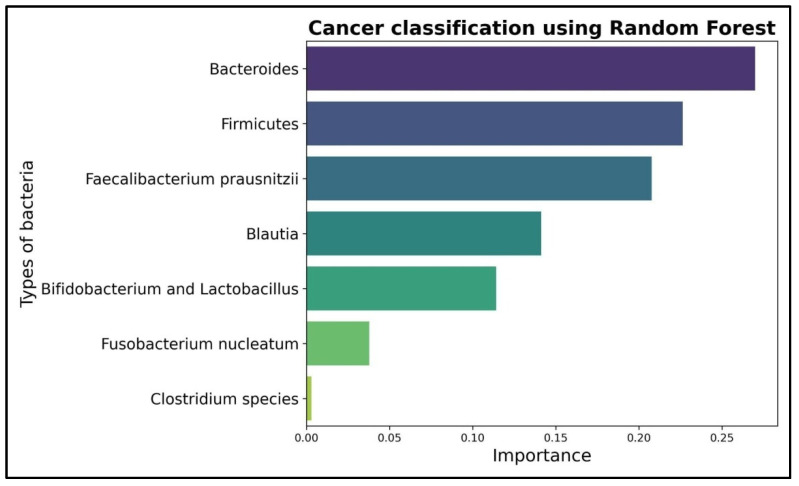
The impact on the decision making process for the remaining analyzed bacteria using a Random Forest AI algorithm.

To conduct a more thorough analysis for the importance of each bacteria in the decision making process, an AI algorithm, namely Random Forest, was trained on the database. The results obtained for the main evaluation criteria—accuracy, precision, recall and f1-score—are all perfect with 100%, which may suggest an overlearning process generated by the small dataset. In the graphic below (Figure 4), the importance of each bacteria using the Random Forest algorithm was measured. The *Clostridium* species has an almost insignificant impact on the decision making process with a coefficient of 0.003 compared to bacteria like *Bacteroides* with a coefficient of 0.27 or *Firmicutes* with a coefficient of 0.226. The impact of either *Bacteroides* or *Firmicutes* is 75 times more important than *Clostridium* species. Therefore, even though it has a high enough Shannon diversity index, a decision to eliminate *Clostridium* species from further analysis was taken.

Breast cancer patients exhibited significantly reduced alpha diversity compared to healthy controls. BRCA carriers showed the lowest microbial diversity among the groups. Overall, BRCA carriers are more susceptible to developing BC compared to BRCA-negative individuals. When BC occurs in BRCA carriers, it tends to be of a higher grade and more aggressive, associated with higher recurrence risk scores and worse survival outcomes. Furthermore, BRCA1 carriers are more likely to develop aggressive BC with a poorer prognosis at a younger age compared to BRCA2 carriers.

The percent for the dominant concentration level of each bacteria calculated in Table 4 for each of the three analyzed groups are determined using the following formula:

$$PercentBacteria=\frac{{max}_{i=1\ldots n}(\sum_{j=1}^{m}\left({bacteriaConcentration}_{j}=i\right))}{m}\;x\;100$$

where n represents the number of concentration levels, in the case of this study 3: low, moderate, and high.

m represents the number of patients in the analyzed group.

*Fusobacterium nucleatum* is commonly overrepresented in breast cancer patients, especially in BRCA carriers, and has been associated with tumor progression, immune modulation, and inflammatory responses. Increased levels of *Blautia* species have been linked to breast cancer, likely due to their role in obesity and hormone metabolism, both of which are risk factors for breast cancer.

*Faecalibacterium prausnitzii*, an anti-inflammatory bacterium, is generally reduced in breast cancer patients, which could contribute to increased inflammation associated with tumorigenesis. Higher microbial diversity was noted in healthy individuals compared to breast cancer patients. Beneficial bacteria such as *Bifidobacterium* and *Lactobacillus*, which contribute to gut and systemic health, were more abundant in healthy controls (Table 4).

One of the main hypotheses of the study is the relationship between gut microbiota and cancer/non-cancer patients; there are 6 bacteria types that are significant for this analysis. To reduce the high dimensionality of the data from a 6-dimensional space, 1 dimension for each bacteria, to a 2-dimensional or 3-dimensional space, an AI technique was chosen that deals with this problem, namely Principal Component Analysis (PCA).

**Figure 5:**
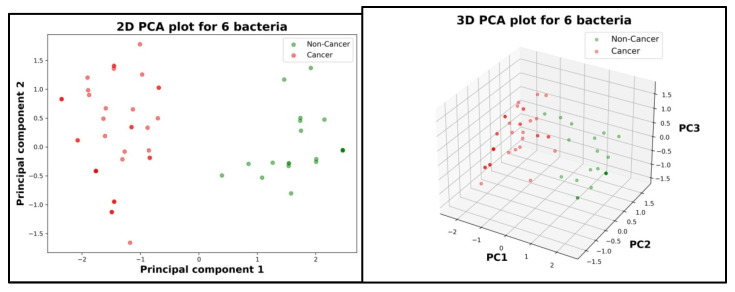
Principal Component Analysis for cancer versus non-cancer patients. Left: the patients scattered in a 2D space. Right: the patients scattered in a 3D space.

By analyzing both 2D and 3D graphics (Figure 5), the two cases are clearly separable so it can be concluded based on a visual representation that there is a relationship between gut microbiota and cancer/non-cancer cases. It can also be observed that cancer patients appear more dispersed than healthy patients, which may suggest a higher variability of gut microbiota for sick people. The linear relationship determined by the PCA algorithm between each PC and all the six bacteria is as follows:

○ For the 2D graphic (Figure 5 Left):

PC1 = −0.272961*Fusobacterium nucleatum + 0.455046*Faecalibacterium prausnitzii − 0.312610*Blautia + 0.442051*Bifidobacterium and *Lactobacillus* − 0.451844*Firmicutes + 0.470242*Bacteroides

PC2 = 0.712624*Fusobacterium nucleatum + 0.201637*Faecalibacterium prausnitzii + 0.531642*Blautia + 0.374008*Bifidobacterium and *Lactobacillus* − 0.154154*Firmicutes + 0.072255*Bacteroides

○ For the 3D graphic (Figure 5 Right):

PC1 = −0.272961*Fusobacterium nucleatum + 0.455046*Faecalibacterium prausnitzii − 0.312610*Blautia + 0.442051*Bifidobacterium and *Lactobacillus* − 0.451844*Firmicutes + 0.470242*Bacteroides

PC2 = 0.712624*Fusobacterium nucleatum + 0.201637*Faecalibacterium prausnitzii + 0.531642*Blautia + 0.374008*Bifidobacterium and *Lactobacillus* − 0.154154*Firmicutes + 0.072255*Bacteroides

PC3 = 0.133008*Fusobacterium nucleatum − 0.131506*Faecalibacterium prausnitzii + 0.144051*Blautia − 0.688158*Bifidobacterium and *Lactobacillus* − 0.520384*Firmicutes + 0.447106*Bacteroides

A statistical experiment consisting of two logistical regression AI models (null model and complete model) was conducted to validate the conclusion drawn from the visual analysis of the results given by PCA. For the null model, a log-likelihood value of 57 was obtained, and for the complete model, the log-likelihood value was 99. A higher value for the log-likelihood score indicates a better model. With a value for the statistical test Likelihood Ratio (LR) of 84 and a *p*-value of 5.32 × 10^−16^, the null hypothesis ($H_{0}$) can be denied with a high degree of confidence, which highlights the existence of a relationship between the main components of the study, namely gut microbiota and cancer.

**Figure 6:**
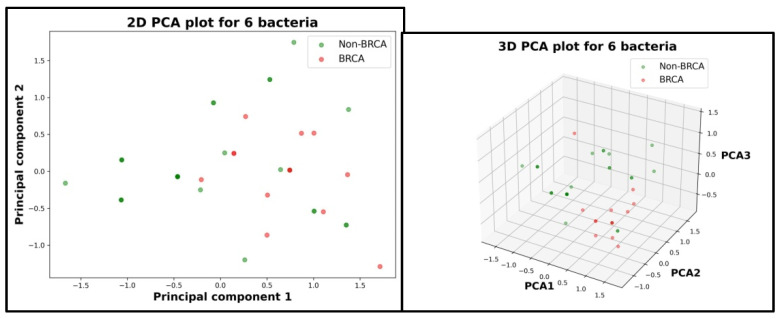
Principal Component Analysis for BRCA versus non-BRCA cancer patients. Left: the patients scattered in a 2D space. Right: the patients scattered in a 3D space.

The same process was followed to analyze if there is a potential relationship between gut microbiota and BRCA gene for cancer patients with the results of PCA presented in the image below (Figure 6).

As it can be seen, this time, the two cases are not linear separable which suggests that gut microbiota has no relationship with the BRCA gene. To strengthen the conclusions of the visual analysis given by PCA, a statistical investigation composed of two logistical regression AI models (null model and complete model) was carried out. For the null model, a log-likelihood value of 44 was obtained, and for the complete model, the log-likelihood value was 50. With a value for the statistical test Likelihood Ratio (LR) of 12 and a *p*-value of 0.062, the null hypothesis ($H_{0}$) is accepted, thus highlighting that, on this database, there is no detectable relationship between gut microbiota and the BRCA gene for cancer patients.

Breast cancer patients showed an overrepresentation of pro-inflammatory and potentially pathogenic bacteria such as *Fusobacterium nucleatum*, while beneficial anti-inflammatory bacteria like *Faecalibacterium prausnitzii* were less abundant compared to healthy controls. This microbial dysbiosis may play a significant role in breast cancer progression and systemic inflammation.

The study found that the gut microbiota of breast cancer patients was significantly more diverse compared to healthy controls. This increased diversity was not observed in healthy women, suggesting that dysbiosis status may influence the relationship between the gut microbiota and breast cancer.

Certain bacteria were found to be more abundant in breast cancer patients, including *E. coli*. The count of *E. coli* in the patients’ stool was significantly elevated, exceeding the normal upper limit (9 × 10^7^ CFU/g). This suggests an overgrowth of *E. coli*, which is associated with the production of biogenic amines and ammonia. These metabolites can induce inflammation and contribute to a toxic environment in the gut. The report shows reduced levels of *Enterococcus* and *Lactobacillus* species, which are crucial for maintaining gut health and preventing colonization by pathogenic bacteria. *Bifidobacterium* levels, although within normal ranges, were at the lower end, indicating a potentially weakened protective flora. An elevated pH indicates a more alkaline environment, often due to the overgrowth of proteolytic bacteria like *E. coli*, which can produce alkaline metabolites.

The report highlights an overgrowth of bacteria capable of producing biogenic amines, such as histamine, which can contribute to inflammatory responses and may exacerbate conditions like cancer by promoting a pro-inflammatory microenvironment.

Certain strains of *E. coli* possess the pks pathogenicity island, which enables them to produce colibactin, a genotoxin that can induce DNA double-strand breaks in host cells. This could contribute to genomic instability and potentially accelerate cancer progression. The overgrowth of *E. coli* can lead to increased levels of lipopolysaccharides (LPS), which trigger systemic inflammation. Chronic inflammation is a well-known risk factor for cancer progression. Beneficial bacteria such as *Lactobacillus* and *Bifidobacterium* are known to produce short-chain fatty acids like butyrate, which possess anti-inflammatory and anti-cancer properties by inhibiting histone deacetylases and modulating immune responses. A reduced presence of these beneficial bacteria can compromise gut barrier integrity, leading to increased gut permeability. This can result in the translocation of bacterial endotoxins into the bloodstream, further promoting systemic inflammation. The overproduction of histamine due to dysbiosis can exacerbate inflammation and potentially contribute to cancer proliferation. Histamine has been shown to influence tumor growth by modulating immune responses and promoting angiogenesis (formation of new blood vessels to feed tumors).

The findings from the stool analysis provide valuable insights into the patients’ gut health, revealing a state of dysbiosis that could be linked to breast cancer diagnosis. Addressing these imbalances through dietary modifications, probiotics, and anti-inflammatory interventions may help improve the patient’s overall health and potentially slow cancer progression.

## 4. Discussion

This study presents a comprehensive characterization of gut microbiota alterations in breast cancer (BC) patients, offering evidence of a distinct microbial signature associated with both cancer presence and BRCA mutation status. Unlike prior research, which often emphasizes general associations between dysbiosis and malignancy, our work integrates advanced computational techniques—such as Shannon diversity index (SDI), Random Forest classification, and Principal Component Analysis (PCA)—to extract and validate microbiota patterns specific to breast cancer and its genetic subtypes. From an initial pool of 18 bacterial taxa, SDI filtering allowed for the identification of seven biologically informative species: *Fusobacterium nucleatum*, *Faecalibacterium prausnitzii*, *Blautia*, *Bifidobacterium*, *Lactobacillus*, and broader groups including *Firmicutes* and *Bacteroidetes*. These taxa were consistently altered in BC patients compared to healthy controls.

Our findings indicate a significantly reduced microbial diversity in BC patients compared to healthy controls, with BRCA mutation carriers exhibiting the lowest diversity among all subgroups. BRCA1 carriers, in particular, were more likely to present triple-negative tumors and malignant lesions diagnosed through biopsy and histopathology, suggesting a more aggressive disease phenotype. The microbial profiles corroborated this observation: 92.31% of BRCA1/2 mutation carriers had low levels of *Faecalibacterium prausnitzii*, a known anti-inflammatory bacterium, while 84.62% exhibited low abundance of beneficial *Bacteroidetes*. Moreover, over 76% of BRCA-positive patients had high levels of *Firmicutes* and 69.23% had elevated *Fusobacterium nucleatum*, highlighting the inflammatory skew of their microbiota.

This aligns with established models that correlate dysbiosis with systemic inflammation and impaired immune regulation—two hallmarks of tumorigenesis. Notably, *F. nucleatum* is known to activate NF-κB signaling via TLR4, thereby promoting chronic inflammation and immune evasion, mechanisms well-documented in colorectal and breast cancers. The pro-inflammatory milieu in BRCA carriers was also evident in their higher prevalence of *Blautia* spp., which are implicated in metabolic dysregulation and obesity, both risk factors for hormone-sensitive BC [6,7,8].

The identification of these bacterial profiles is further supported by machine learning outputs. Random Forest analysis assigned disproportionately high weights to *Bacteroides* (0.27) and *Firmicutes* (0.226) compared to minimal contributions from *Clostridium* species (0.003), leading to its exclusion from subsequent analysis. PCA analyses revealed a clear visual separation between cancer and non-cancer cohorts, supporting the hypothesis of a microbiota–tumor axis. Statistical validation through logistic regression reinforced these observations (log-likelihood ratio = 84, *p* = 5.32 × 10^−16^), providing strong evidence for a relationship between microbiota composition and cancer status.

In contrast, microbiota differences between BRCA-positive and BRCA-negative cancer patients were not linearly separable by PCA, and logistic regression analysis failed to reject the null hypothesis (*p* = 0.062). While this result may reflect a true biological null, it is more likely explained by the small number of BRCA carriers (*n* = 13), limiting statistical power. Still, the observed trends—such as lower *Bifidobacterium* and *Lactobacillus* levels and higher microbial variability among BRCA carriers—suggest a potential interaction between genetic susceptibility and microbial ecology that warrants further investigation.

One of the most salient findings is the elevated presence of *E. coli*, particularly strains potentially harboring the pks pathogenicity island capable of producing colibactin. Although not sequenced directly, the abnormally high stool counts (exceeding 9 × 10^7^ CFU/g) imply potential genotoxic capacity. This microbial component could contribute to DNA double-strand breaks and genomic instability in host tissues, a mechanism that may act synergistically with inherited BRCA mutations to accelerate carcinogenesis. Further, elevated stool pH and depletion of protective flora such as *Lactobacillus* and *Enterococcus* corroborate a shift toward proteolytic and alkali-producing bacterial populations, conducive to a toxic and pro-inflammatory gut environment.

The hormonal axis further contextualizes the observed dysbiosis. Several gut bacteria, particularly within the *Firmicutes* phylum, express β-glucuronidase, an enzyme that reactivates conjugated estrogens in the gut, increasing their systemic bioavailability. This process may exacerbate the risk in estrogen receptor-positive BC, especially in postmenopausal women who rely on peripheral aromatization for estrogen production. In our dataset, *Firmicutes* were significantly overrepresented in both BRCA and non-BRCA BC patients, while *Bacteroidetes* and SCFA-producing taxa were consistently depleted, pointing to impaired estrogen metabolism and weakened anti-inflammatory buffering [9,10].

From a translational perspective, the demonstration that microbiota profiles can stratify cancer risk suggests potential for non-invasive diagnostic biomarkers and therapeutic targets. Microbiota-modulating strategies—such as dietary interventions, prebiotics, probiotics, and even fecal microbiota transplantation—could augment existing treatment modalities by restoring microbial balance, improving immune function, and potentially reducing tumor-promoting inflammation [11,12]. Notably, the PCA model’s ability to classify cancer status visually and statistically, using only microbiota data, reinforces the potential utility of these findings in early diagnostic frameworks.

Nevertheless, this study is constrained by several limitations. The modest sample size limits generalizability and statistical power, particularly for subgroup analyses like BRCA vs. non-BRCA comparisons. Additionally, the cross-sectional nature precludes causal inferences, and although patients were treatment-naïve and antibiotic-free, unmeasured environmental or dietary confounders may still have influenced microbiota composition. Lastly, despite sophisticated modeling approaches, the study lacks longitudinal data that would clarify whether observed dysbiosis is a driver or consequence of disease. Future studies should adopt prospective designs with repeated sampling and include functional microbial analysis, such as metagenomics or metabolomics, to better delineate causal mechanisms.

The findings of this study, which demonstrate a distinct gut microbial signature in breast cancer patients characterized by elevated levels of *Firmicutes* and *Fusobacterium nucleatum*, provide a critical foundation for exploring the underlying mechanisms. These bacterial taxa are increasingly recognized for their roles in promoting local and systemic inflammation [13], which is a known driver of carcinogenesis [14]. For instance, *Fusobacterium nucleatum* possesses adhesins that allow it to attach to epithelial cells and activate pro-inflammatory signaling pathways, such as NF-κB, through the stimulation of Toll-like receptor 4 (TLR4) on immune cells. This activation leads to the production of cytokines, which can create a pro-tumorigenic microenvironment and contribute to the chronic inflammation observed in various cancers, including breast cancer [15].

Beyond inflammation, the gut microbiota can significantly influence host hormonal metabolism, a central pathway in the etiology of hormone-receptor-positive breast cancer. Bacteria within the gut, particularly certain species of *Firmicutes* and other phyla, produce enzymes such as β-glucuronidase [16]. This enzyme deconjugates estrogen metabolites that have been excreted into the bile, allowing them to be reabsorbed into the enterohepatic circulation [17]. This process, if dysregulated, can lead to increased systemic estrogen levels, which can stimulate the proliferation of estrogen-sensitive breast cancer cells. The observed shift in the microbial community in our breast cancer cohort may therefore contribute to altered hormonal homeostasis, providing a plausible link between the microbial signature and disease pathogenesis [18,19,20].

The potential for direct genotoxic effects of the microbiota also warrants discussion. As highlighted in the literature, certain strains of *E. coli* can produce genotoxins like colibactin. This compound is known to induce DNA double-strand breaks in host cells, a critical event in the initiation of cancer. While this study did not specifically quantify these toxins, the presence of certain bacterial species in the breast cancer group, as identified by our analysis, suggests that a microenvironment conducive to genotoxic activity may be present. This mechanism, combined with chronic inflammation and altered hormonal metabolism, offers a multifaceted explanation for how a dysbiotic gut microbiota could contribute to the development and progression of breast cancer.

### 4.1. Data Analysis and Interpretation

The normal distribution is one of the most commonly used in modern science because many statistical methods like Pearson coefficient, linear regression, ANOVA test, chi-squared test, *t* test, and Fisher test need the data to follow a normal/Gaussian distribution. The main parameters for a normal distribution are the mean and the standard deviation. If a set of data follows a normal distribution, then it should be nearly symmetrical to the left and right of the mean value, approximately 68% of the data should be in the interval mean ± standardDeviation, and approximately 95% of the data should be in the interval mean ± 2*standardDeviation [21]. In our case, the mean value for age is 59.84 years and the standard deviation is 11.64. For a good evaluation of the age distribution of the patients, we employed a few classical methods like histogram, quartile to quartile plot, and one normal distribution test, namely Shapiro–Wilk.

An SDI technique was employed on the data with the goal to eliminate the bacteria that are almost constant for all the patients, regardless of them being having or not having cancer. If a bacterium has a low value for the Shannon index, then, depending on a certain threshold, we can decide to eliminate it. So far, there is no universal and standardized threshold for the index, but studying the heatmap for all bacteria, an obvious difference can be seen between certain groups.

For a more in-depth analysis of the importance of all bacteria in the decision process, we employed an AI algorithm, namely Random Forest. The idea of using this approach comes more from the desire to analyze the impact of one certain bacteria, *Clostridium* species, which has a medium value for the Shannon diversity index, rather than to evaluate the performance of the decision process of an AI algorithm. We did not take into consideration the AI performance in classifying the cancer and non-cancer patients, not because of the lack of interest in integrating new and emerging technologies in the decision process, but due to the lack of a bigger dataset; this task is too easy for a complex AI architecture like Random Forest.

A good and simple way to understand more from the combination of these bacteria in cases of cancer and non-cancer patients is to make a form of visual representation for all the bacteria. This would be a very good idea if the data were two-dimensional (two bacteria analyzed) or three-dimensional (three bacteria analyzed) but a higher dimensionality is hard to impossible for humans to understand. In order to solve this issue, we chose to make use of a component of artificial intelligence that deals with this problem, namely Principal Component Analysis (PCA). PCA is a technique of dimensional reduction that can transform a six-dimensional space into a two or three-dimensional space by creating principal components (PC) based on the six bacteria. Based on the graphics produced by PCA, a clear separation between cancer and non-cancer patients can be observed. These conclusions are further strengthened by a statistical comparison of two regression models, one that trains on the data and the other that does not see the data.

### 4.2. Limitations and Future Works

This study identifies a distinct microbial signature in breast cancer patients, characterized by an overrepresentation of certain *Firmicutes* and a reduction in beneficial *Bacteroides* compared to healthy individuals. The finding that the microbiota can be used to distinguish cancer from non-cancer patients is visually supported by the PCA plots. This investigation, while providing novel insights into the association between gut microbiota and breast cancer, is subject to several methodological limitations that warrant consideration and provide clear directions for future research.

The study’s primary limitation is the relatively modest sample size (*n* = 99), which restricted the statistical power and necessitates a cautious interpretation of the findings. The observed correlations, while biologically plausible, should be considered exploratory and hypothesis-generating. Future research should prioritize the recruitment of a significantly larger, geographically and ethnically diverse cohort to validate these preliminary findings and enhance their generalizability.

Another significant limitation of the study imposed by the dimensionality of the database is the impossibility of a more in-depth analysis for BRCA vs. non-BRCA cases. This is a result from the fact that a further division by any criteria of the databases, especially for the 13 BRCA cases, would fragment too much the clusters that could lead to the creation of subgroups with 4–5 cases. In future research, when the databases have grown significantly, further exploration between key factors like histotype, phenotype, stage and age for the BRCA vs. Non-BRCA cases should be carried out.

While this study establishes a compelling association between a distinct microbial signature and breast cancer, it is inherently cross-sectional and cannot infer causality. The identified differences in microbiota composition could be a consequence, rather than a cause, of breast cancer or its treatment. To address this, future investigations should adopt a longitudinal study design. A prospective cohort study, following healthy individuals over time and collecting serial fecal samples, would be instrumental in determining whether these microbial signatures precede the diagnosis of breast cancer, thereby strengthening the argument for a causal role. This would also allow for the assessment of dynamic changes in the microbiota throughout the course of the disease and its treatment, offering a more comprehensive understanding of its role in breast cancer pathogenesis.

## 5. Conclusions

The human gut microbiota represents a complex and dynamic ecosystem that exerts a profound influence on host physiology, extending far beyond its fundamental role in digestion. Through the fermentation of non-digestible dietary fibers, the microbiota produces key metabolites, such as short-chain fatty acids (SCFAs), which serve as an energy source for colonocytes and play a crucial role in regulating host metabolism and appetite. Furthermore, this microbial community is a central modulator of the host immune system, actively shaping both innate and adaptive immunity. It is instrumental in training immune cells, maintaining the integrity of the intestinal epithelial barrier, and fine-tuning inflammatory and anti-inflammatory pathways. A state of imbalance, or dysbiosis, in this microbial community can disrupt these critical functions, leading to systemic inflammation and creating a permissive environment for the development and progression of various pathologies, including chronic inflammatory diseases and different forms of cancer [15,22,23]. Therefore, understanding these intricate, bidirectional interactions is essential for unraveling the specific mechanisms by which microbial dysbiosis contributes to disease pathogenesis.

There is evidence to suggest that the composition of the breast microbiota differs between healthy individuals and those with breast cancer [24,25,26,27]. How this microbiota might influence breast cancer development and progression is not fully understood, but several mechanisms have been proposed. These include chronic inflammation induced by harmful bacteria, interference with the local immune response, metabolic changes, and influence on hormone levels. For instance, some bacteria can produce enzymes that metabolize estrogen, which can influence breast cancer risk and progression [28]. While this is supported by recent studies [29], it remains an assumption requiring further validation. BRCA mutation carriers may exhibit more pronounced microbial dysbiosis due to the potential influence of genetic predisposition on systemic inflammation and immune modulation.

This article sets the stage for an in-depth exploration of the microbiota’s role in breast cancer, highlighting the potential for novel insights and therapeutic strategies. It emphasizes the importance of considering microbiota as a significant player in breast cancer pathogenesis and progression, alongside genetic, hormonal, and environmental factors. The gut microbiota presents a novel avenue for understanding and potentially managing breast cancer. While current evidence is promising, further research is needed to elucidate the exact mechanisms and develop targeted interventions.

## Figures and Tables

**Table 1:** Key statistics regarding the number of subjects studied.

Group	Number of Participants	Urban	Rural
**Total Participants**	99	38	19
**Breast Cancer Patients**	57	38	19
BRCA Mutation Carriers	13	8	5
Non-BRCA Carriers	44	30	14
**Healthy Controls**	38	28	14

**Table 2:** Profiles of the two subgroups of BRCA carriers (Legend: BIO = Biopsy; CL = Clinical diagnosis; DCIS = Ductal carcinoma in situ; F-C Ch = Fibro-cystic change; HP = Histopathology; IDC = Invasive ductal carcinoma; ILC = Invasive lobular carcinoma; L-A = Luminal A type; L-B = Luminal B type; M = Malignancies; NCO = Not carried out; NN = Non-neoplastic lesions; NOS = Not otherwise specified; OP = Surgical procedure; T-N = Triple-negative).

Groups		BRCA1 Carriers	BRCA2 Carriers
Cases		9	4
Age	**≤40 years**	3	0
	**>40 years**	6	4
Family history of breast cancer	**Yes**	7	0
	**No**	2	4
Diagnostic procedure	**CL**	1	0
	**CL + BIO**	6	3
	**OP + HP**	2	1
Histogenetic type	**NOS**	1	1
	**F-C Ch**	0	0
	**M**	8	3
Histopathological diagnosis	**NCO**	1	0
	**NN**	0	0
	**DCIS**	0	0
	**IDC**	7	3
	**ILC**	1	1
Molecular classification	**NCO**	1	0
	**L-A**	0	3
	**L-B**	2	0
	**HER2+**	0	0
	**T-N**	6	1

**Table 3:** Profiles of the non-BRCA breast cancer patients (Legend: BIO = Biopsy; CL = Clinical diagnosis; DCIS = Ductal carcinoma in situ; F-C Ch = Fibro-cystic change; HP = Histopathology; IDC = Invasive ductal carcinoma; ILC = Invasive lobular carcinoma; L-A = Luminal A type; L-B = Luminal B type; M = Malignancies; NCO = Not carried out; NN = Non-neoplastic lesions; NOS = Not otherwise specified; OP = Surgical procedure; T-N = Triple-negative).

Groups		Non-BRCA Breast Cancer Patients
Cases		44
Age	**≤40 years**	1
	**>40 years**	43
Family history of breast cancer	**Yes**	2
	**No**	42
Diagnostic procedure	**CL**	0
	**CL + BIO**	44
	**OP + HP**	0
Histogenetic type	**NOS**	21
	**F-C Ch**	0
	**M**	23
Histopathological diagnosis	**NCO**	0
	**NN**	0
	**DCIS**	13
	**IDC**	16
	**ILC**	15
Molecular classification	**NCO**	0
	**L-A**	10
	**L-B**	18
	**HER2+**	11
	**T-N**	5

**Table 4:** Bacteria distribution in subjects.

Group	Breast Cancer Patients (BRCA Carriers)	Breast Cancer Patients (Non-BRCA Carriers)	Healthy Controls
***Fusobacterium nucleatum***	High 69.23%	Moderate 75.00%	Low 83.33%
***Faecalibacterium prausnitzii***	Low 92.31%	Low 81.82%	High 83.33%
**Blautia species**	High 84.62%	Moderate 75.00%	Low 95.24%
**Bifidobacterium and Lactobacillus**	Low 61.54%	Low 88.64%	High 88.10%
**Firmicutes (Clostridium clusters XIVa, IV)**	High 76.92%	High 77.27%	Low 90.48%
**Bacteroidetes (Bifidobacterium, Coprococcus, etc.)**	Low 84.62%	Low 86.36%	High 90.48%
Key Observation	Overrepresentation of pro-inflammatory bacteria; dysbiosis linked to tumor progression	Moderate dysbiosis with increased *Firmicutes* and reduced beneficial bacteria	Healthy microbiota composition with higher beneficial bacteria and diversity

## Data Availability

The original contributions presented in this study are included in the article. Further inquiries can be directed to the corresponding author.
